# Frequency of ERBB2-Low Expression in Endometrial Cancer

**DOI:** 10.1001/jamaoncol.2024.3660

**Published:** 2024-09-05

**Authors:** Camilla Krakstad, Hege F. Berg, Kristina Lindemann, Mari Kyllesø Halle

**Affiliations:** 1 Department of Obstetrics and Gynaecology, Haukeland University Hospital, Bergen, Norway; 2 Centre for Cancer Biomarkers, Department of Clinical Science, University of Bergen, Bergen, Norway; 3 Department of Gynecological Oncology, Oslo University Hospital, Oslo, Norway; 4 Faculty of Medicine, Institute for Clinical Medicine, University of Oslo, Oslo, Norway

## Abstract

This study reanalyzes epidermal growth factor receptor 2 (ERBB2) data from a previous study to characterize patient subgroups with ERBB2-low tumors.

The epidermal growth factor receptor 2 (ERBB2, formerly HER2)–targeting antibody drug conjugates (vic-)trastuzumab duocarmazine and trastuzumab deruxtecan have recently been tested in endometrial cancer (EC), including patients with ERBB2 immunohistochemistry score 1+ and 2+–expressing tumors. Interim results^1^ show response rates in patients with cervical (50.0%), endometrial (57.5%), and ovarian cancer (45.0%). These response rates suggest practice-changing efficacy and support further investigation in phase 3 trials in EC. We therefore reanalyzed ERBB2 data previously reported^2^ and added information on all ERBB2 scoring categories and molecular subtype, aiming to characterize patient subgroups also with ERBB2-low tumors (1+ and 2+).

## Methods

For this diagnostic study, data were collected prospectively from May 5, 2001, to January 21, 2015. Data analysis was conducted in spring 2024. The reanalysis of data was approved by the local regional ethics committee of the original study. Details on the population-based patient cohort, ethical approvals, and staining and scoring of ERBB2 in 4 categories (HercepTest [Agilent] score 0, 1+, 2+, and 3+) have been described previously.^2^ Molecular classes were defined by molecular marker detection in the following order: (1) DNA polymerase epsilon (*POLE)* alteration, (2) mismatch repair deficiency, (3) p53 wild-type/copy-number low, or (4) p53 aberrant/copy-number high. The data were analyzed using SPSS version 29 (IBM) by the Pearson χ^2^ test and the Kruskal-Wallis test. All *P* values are 2-sided and considered statistically significant at <.05.

## Results

A total of 87% (687 of 790) of all lesions were positive when including all ERBB2 of 1+ or higher while 56% were 2+ or higher (Table). In contrast, only 17% were ERBB2-positive according to a 3+ cutoff. While 3+ tumors were associated with high patient age, patients with 1+ and 2+ tumors had similar age as patients with no ERBB2 expression. Among the endometrioid tumors, most were 1+ or 2+ (74%), while for serous and clear cell tumors, most lesions were 2+ (78%) or 3+ (66%). Among the more aggressive EC subtypes, 86% of the grade 3 endometrioid tumors, 95% of serous lesions, 88% of carcinosarcomas, 89% of clear cell, and 77% of undifferentiated were ERBB2-positive (≥1+).

**Table.** Clinicopathological and Molecular Characteristics for Patients With EC by HercepTest Score

Characteristic	HercepTest score, No. (%)^a^				*P* value^b^
	0	1+	2+	3+	
No./total No. (%)	103/790 (13)	244/790 (31)	309/790 (39)	134/790 (17)	NA
Median age (range), y (N = 790)	66 (38-88)	64 (25-88)	64 (28-93)	70 (39-93)	<.001^c^
Histologic type (N = 790)					
Endometrioid	86 (13)	210 (33)	265 (41)	80 (13)	<.001
Serous	5 (7)	11 (15)	21 (29)	35 (49)	
Clear cell	4 (13)	7 (23)	10 (33)	9 (30)	
Carcinosarcoma	5 (15)	11 (32)	10 (29)	8 (23)	
Undifferentiated or other	3 (23)	5 (39)	3 (23)	2 (15)	
Grade-endometrioid tumor (n = 641)					
Grade 1	40 (14)	91 (31)	130 (45)	29 (10)	.08
Grade 2	30 (14)	78 (35)	89 (40)	26 (12)	
Grade 3	15 (14)	36 (33)	36 (33)	23 (21)	
FIGO stage (n = 790)					
Stage I	76 (13)	193 (32)	239 (40)	94 (16)	.30
Stage II	6 (9)	19 (30)	28 (44)	11 (17)	
Stage III	14 (16)	22 (24)	34 (38)	20 (22)	
Stage IV	7 (21)	10 (29)	8 (23)	9 (26)	
Metastatic or recurrent disease (n = 789)					
No recurrence	79 (13)	192 (31)	247 (41)	92 (15)	.12
Metastatic at primary	9 (16)	11 (20)	21 (38)	14 (26)	
Later recurrence	15 (12)	40 (32)	41 (33)	28 (23)	
Molecular subtype (n = 633)					
POLE-altered (8%)	7 (13)	16 (30)	23 (43)	7 (13)	<.001
MMR deficient (24%)	23 (15)	50 (33)	65 (42)	16 (10)	
Copy-number low (54%)^d^	38 (11)	117 (34)	150 (44)	38 (11)	
Copy-number high (13%)	6 (7)	16 (19)	25 (30)	36 (43)	

Abbreviations: EC, endometrial cancer; FIGO, Federation International of Gynecology and Obstetrics; MMR, mismatch repair; NA, not applicable; POLE, DNA polymerase epsilon.

^a^ HercepTest (Agilent) scores 0 and 1+ are negative; 2+ is weakly positive; and 3+ is positive.

^b^ All *P* values represent χ^2^ test, except as noted.

^c^ Kruskal-Wallis test.

^d^ Missing POLE status (n = 87).

Metastatic disease at diagnosis or later recurrence was seen in 31% of patients with 3+ tumors, 20% with 2+ tumors, 21% with 1+ tumors, and 23% with ERBB2-negative tumors. A total of 142 of 790 patients had tissue available from one or more corresponding metastases, and 78% of the metastatic lesions were ERBB2 of 1+ or higher. The concordance between ERBB2 expression in paired primary and metastatic EC was 64% (91 of 142 patients). Copy-number high tumors have the highest prevalence of 3+ tumors. However, a substantial number of the intermediate-risk lesions were ERBB2 1+ or ERBB2 2+.

## Discussion

This study provides additional data on prevalence of ERBB2-low (1+ and 2+) expression from a large population-based endometrial cancer series. This study is limited by the lack of treatment with ERBB2-targeting ADCs. In this study, positive ERBB2 tumors were found in all EC clinicopathologic and molecular subgroups, also in metastases or recurrent lesions. To date, few studies have presented the prevalence of ERBB2 1+ and 2+ in EC, ranging from 64% to 82% in serous tumors^3,4^ and 70% (≥1+) in stage III, IV, or recurrent tumors,^5^ slightly lower than we report here. In contrast with breast and gastric cancer, no established ERBB2 testing guidelines exist for EC. Lack of validated ERBB2 tests in EC is a challenge for submission of future trials to regulatory authorities and potentially also for subsequent reimbursement in clinical practice, and interpretation of results required in studies may differ from what is established in standard of care. Exploring response relative to ERBB2 expression levels in concluded trials prior to initiating large scale phase 3 trials is crucial to make this strategy accessible for all patients who could potentially benefit. By presenting extended data for ERBB2 levels in this large population-based EC cohort, we show that initiatives investigating ERBB2 targeting strategies in EC may have practice-changing implications across molecular groups.

## Notes

Supplement.
Data Sharing Statement
