# Supplementary material for: Frequency of ERBB2-Low Expression in Endometrial Cancer
Source: JAMA Oncol. 2024 Sep 5;10(11):1587–8. doi: 10.1001/jamaoncol.2024.3660 (PMC11378060; doi:10.1001/jamaoncol.2024.3660)
Supplement: Supplement. — Data Sharing Statement [file jamaoncol-e243660-s001.pdf]

Data Sharing Statement

Krakstad. Frequency of ERBB2-Low Expression in Endometrial Cancer. *JAMA Oncol*.
Published September 05, 2024. doi:10.1001/jamaoncol.2024.3660

Data
Data available: No

Additional Information
Explanation for why data not available: Data access can be granted after contact with the corresponding author, pending approval by the local ethical committee
